# Non-Overlapping Distributions and Functions of the VDAC Family in Ciliogenesis

**DOI:** 10.3390/cells4030331

**Published:** 2015-07-31

**Authors:** Shubhra Majumder, Ayla Cash, Harold A. Fisk

**Affiliations:** Department of Molecular Genetics, The Ohio State University, 484 W. 12th Avenue, Columbus, OH 43210-1292, USA; E-Mails: majumder.7@osu.edu (S.M.); aaecash@gmail.com (A.C.)

**Keywords:** centrosome, cilia, ciliogenesis, VDAC, mitochondria

## Abstract

Centrosomes are major microtubule-organizing centers of animal cells that consist of two centrioles. In mitotic cells, centrosomes are duplicated to serve as the poles of the mitotic spindle, while in quiescent cells, centrosomes move to the apical membrane where the oldest centriole is transformed into a basal body to assemble a primary cilium. We recently showed that mitochondrial outer membrane porin VDAC3 localizes to centrosomes where it negatively regulates ciliogenesis. We show here that the other two family members, VDAC1 and VDAC2, best known for their function in mitochondrial bioenergetics, are also found at centrosomes. Like VDAC3, centrosomal VDAC1 is predominantly localized to the mother centriole, while VDAC2 localizes to centriolar satellites in a microtubule-dependent manner. Down-regulation of VDAC1 leads to inappropriate ciliogenesis, while its overexpression suppresses cilia formation, suggesting that VDAC1 and VDAC3 both negatively regulate ciliogenesis. However, this negative effect on ciliogenesis is not shared by VDAC2, which instead appears to promote maturation of primary cilia. Moreover, because overexpression of VDAC3 cannot compensate for depletion of VDAC1, our data suggest that while the entire VDAC family localizes to centrosomes, they have non-redundant functions in cilogenesis.

## 1. Introduction

Centrosomes that serve as the major microtubule-organizing centers (MTOC) in animal cells contain two structurally distinguishable centrioles surrounded by pericentriolar matrix (PCM). The oldest of the centriole pair, the “mother”, contains appendages, while the other centriole (the “daughter”) does not. During the S-phase of the cell cycle, a new centriole is assembled at the proximal end of each existing centriole. Once duplicated, centrosomes are separated and moved to opposite sides of a cell in late G2-phase in order to serve as the poles of the mitotic spindle [1].

Another important role of centrioles is to assemble cilia (or flagella) that are microtubule-based and membrane-ensheathed projections from the cell surface [2]. Of the two major types of cilia [3], motile cilia are found at specialized epithelial layers where they regulate directional fluid flow. In contrast, non-motile primary cilia that serve as cellular antenna by transducing physiological signals are found in almost all types of animal cells, commonly when the cells are quiescent or differentiated [4]. During cilia assembly, the distal end of the mother centriole becomes encapsulated by golgi-derived vesicles and docks at the apical membrane where it is transformed into a basal body [5]. Ciliary proteins and membrane components assemble on the basal body, and through the activity of Intraflagellar transport (IFT), the centriolar axoneme is elongated and matures into a primary cilium [6]. Cilia are resorbed when cells re-enter S-phase, thereby allowing centrioles to replicate to maintain centriole homeostasis in cycling cells [7,8]. Several core centriolar proteins, components of centriolar satellites, and other regulatory proteins including IFT proteins have been identified as crucial for cilia assembly, maturation and function [9,10,11,12]. Comparatively few proteins have been identified that may negatively regulate ciliogenesis [7,13]. Moreover, the understanding of the intricate mechanism(s) regulating ciliary disassembly and the interconversion between centrioles and basal bodies remained largely unrevealed.

Voltage dependent anion channel (VDAC) proteins are channel-forming proteins of the outer mitochondrial membrane (OMM) and have mostly been studied for their gate-keeping functions that regulate the exchange of metabolites, ions and ATP between mitochondria and cytoplasm (reviewed in [14]). Humans contain three VDACs, VDAC1, VDAC2 and VDAC3, which share significant homology (roughly 60%–70%) in their sequence. Structural studies indicated that VDAC1 likely adopts a β-barrel structure at the OMM [15,16,17], and based on their homology, the other two VDACs are also predicted to have similar three-dimensional conformation [18]. Importantly, several reports suggest that VDAC1 may localize at cellular locations other than mitochondria, and may perform functions other than bioenergetics [19,20,21,22]. For example, VDAC2 and VDAC3 were identified in sperm outer dense fibers (ODF), a non-membranous cytoskeletal compartment important for flagellar motility [23]. In fact, we have recently identified a centrosome-associated pool of VDAC3, and an interaction of VDAC3 with Mps1, a protein kinase that plays important roles in centriole assembly during S-phase [24,25] and spindle assembly checkpoint during mitosis [26,27]. VDAC3 is important to recruit Mps1 to centrosomes during S-phase, and centriole assembly is inhibited in VDAC3-depleted cells [28]. Interestingly, we also found that depletion of VDAC3 in human RPE1 cells led to a striking increase in the number of cells containing cilia in a non-quiescent population [29]. This aberrant ciliogenesis phenotype was not due to inhibition of the mitochondrial function of VDAC3 since perturbation of the mitochondrial activity of VDAC3 or general disruption of mitochondrial bioenergetics did not affect cilia assembly. Mps1-depleted cells also aberrantly formed cilia, and were unable to disassemble cilia upon release from serum starvation, suggesting that a novel Mps1-VDAC3 module may negatively regulate ciliogenesis [29].

Here, we asked if VDAC1 and VDAC2 also localize to centrosomes, and whether they play any role in ciliogenesis. Our study shows that both VDAC1 and VDAC2 are found at centrosomes. We also show that VDAC1 negatively regulates ciliogenesis similar to VDAC3, but that the roles of VDAC1 and VDAC3 in ciliogeneisis are non-redundant. In addition, the ability to negatively regulate ciliogenesis is not shared by VDAC2, which appears to be important for maturation of primary cilia.

## 2. Experimental Section

### 2.1. Plasmids

Previously described plasmids used for this study are as follows: Bacterial expression constructs; pHF270 (6xHis-VDAC1), pHF271 (6xHis-VDAC2), pHF272 (6xHis-VDAC3); mammalian expression constructs pHF286 (GFP), pHF279 (GFP-VDAC3), pHF283 (GFP-sirVDAC3) [28,29]. Plasmids created for this study are as follows: GFP-tagged mammalian expression constructs; pHF307 (GFP-VDAC1), pHF308 (GFP-VDAC2) and pHF309 (GFP-sirVDAC1). Plasmids pHF307 and pHF308 were created using PCR to flank the VDAC1 and VDAC2 open reading frames from pHF270 and pHF271 respectively with KpnI and XbaI for cloning into pHF286 using Infusion HD cloning kit (Clontech). The siRNA resistant VDAC1 expression construct pHF309 was created by site-directed mutagenesis of pHF308, using the QuikChange XL Site-Directed Mutagenesis Kit (Stratagene). The sequences of PCR primers used in this study are provided in supplemental Table S1. The identity of all constructs was verified by sequence analysis.

### 2.2. Cell Culture

hTERT-RPE1 (RPE1) cells were cultured in DME/F-12 (1:1) media (Hyclone) supplemented with 10% FBS (Atlanta Biologicals), 100 U/mL penicillinG and 100 μg/mL streptomycin (Hyclone) at 37 °C in a humidified chamber in the presence of 5% CO_2_. To identify cells in S-phase, cells were either incubated with EdU (10 μM; Invitrogen) or BrdU (40 μM; Sigma) for 4 h before fixation. For serum starvation, RPE1 cells were incubated in serum and antibiotic free DME/F12 (1:1) for 24 or 48 h.

### 2.3. DNA and siRNA Transfections

Mammalian constructs were transfected using FuGENE 6 (Promega). Silencer Select siRNAs directed against VDAC1 (siVDAC1: nucleotides 736-754), VDAC2 (siVDAC2: nucleotides 604-622), and Stealth VDAC3 (siVDAC3: nucleotides 330-354; [28]) were obtained from Invitrogen and Lamin A/C siRNA (siControl) obtained from Dharmacon. siVDAC1 and siVDAC2 were used at a final concentration of 20 nM, and siVDAC3 at a final concentration of 40 nM for transfection using Lipofectamine RNAiMAX (Invitrogen).

### 2.4. Cytology

Antibodies and working dilutions for indirect immunofluorescence (IIF) were as follows: GTU-88 mouse anti-γ-tubulin, 1:200 (Sigma); rabbit anti-γ-tubulin, 1:200 (Sigma); goat anti-γ-tubulin, 1:50 (Santa Cruz Biotechnology); mouse anti-acetylated tubulin (Ac-Tub), 1:1000 (Sigma); GT335 mouse anti-glutamylated tubulin, 1:1000 (Sigma); DM1A mouse anti-α-tubulin, 1:1000 (Sigma); rabbit anti-VDAC3, 1:50 (Aviva Systems Biology); rabbit anti-Arl13B, 1:100 (Proteintech Group); 3E6 mouse anti-GFP, 1:250 (Invitrogen); rabbit anti-Cep135, 1:500 (Abcam); rabbit anti-VDAC1, 1:100 (Cell Signaling); goat anti-VDAC2, 1:100 (Abcam); rabbit anti-VDAC, 1:100 (Calbiochem), rabbit anti-GFP, 1: 1000 (Abcam); mouse anti-Cenexin, 1:500 (Abcam); mouse anti-human mitochondria, 1:200 (Millipore); rabbit anti-CoxIV, 1:250 (Cell Signaling); rabbit anti-PCM1, 1:500 (Cell Signaling). Secondary antibodies for IIF were donkey anti-rabbit, donkey anti-mouse, donkey anti-goat, or goat anti-rat conjugated to Alexa 350 (1:200), Alexa 488 (1:1000), Alexa 594 (1:1000; all from Invitrogen), and donkey anti-goat, donkey anti-mouse or donkey anti-rabbit conjugated to IRDye 800 (1:200; all from Rockland). DNA was stained with Hoechst 33342 (Sigma). Cells were fixed with either PBS containing 4% formaldehyde (Ted Pella) and 0.2% Triton X-100 for 10 min at room temperature, or in methanol at −20 °C for 10 min. For assessing mitochondrial function, cells were incubated with 100 μM MitotrackerRed (Invitrogen) for 1 h, and then in MitotrackerRed-free medium for 30 min prior to fixation in formaldehyde. For visualizing BrdU, cells fixed in methanol were stained with primary and secondary antibodies to cellular antigens, fixed again in methanol, treated with 2 N HCl for 30 min at room temperature, followed by staining with anti-BrdU antibody as previously described [28]. Click-iT EdU Cell Proliferation kit (Invitrogen) was used according to manufacturer’s instruction to visualize EdU-positive cells. The centrosomal level of VDAC1 was measured as a ratio of the total fluorescence signal of VDAC1 to that of γ-tubulin at centrosomes using our recently described quantitative IIF technique [30]. BrdU-positive cells from both samples were imaged under identical conditions for the analysis. All images were acquired at ambient temperature using an Olympus IX-81 microscope, with a 63X or 100X Plan Apo oil immersion objective (1.4 numerical aperture), and a QCAM Retiga Exi FAST 1394 camera, and analyzed using the Slidebook software package (Intelligent Imaging Innovations).

### 2.5. Immunoblotting

Cells were lysed in either lysis buffer (50 mM Tris-HCl pH8.0, 150 mM NaCl, and 1% NP-40) or RIPA buffer (50 mM Tris-HCl pH8.0, 150 mM NaCl, and 1% TritonX-100, 0.25% Na-Deoxycholate, 0.1% SDS). Protein concentrations of cellular lysates were measured using BCA protein assay kit (Pierce). Antibodies for immunoblot analysis were: 1:20,000 DM1A mouse anti α-Tubulin (Sigma); 1:1000 rabbit anti-VDAC3 (Aviva Systems Biology); 1:20,000 mouse anti-GAPDH (Sigma); 1:1000 mouse anti-γ-tubulin (Sigma); rabbit anti-γ-tubulin, 1:1000 (Sigma); 1:1000 rabbit anti-VDAC1 (Cell Signaling); 1:2000 goat anti-VDAC2 (Abcam); 1: 500 rabbit anti-VDAC (Calbiochem), 1:2000 rabbit anti-CoxIV (Cell Signaling); 1:2000 rabbit anti-Nucleolin (Sigma). Secondary antibodies for use with the Odyssey imaging system (Li-Cor) were Alexa680-conjugated donkey anti-mouse/rabbit (Invitrogen) and IRDye800-conjugated donkey anti-mouse/rabbit/goat (Rockland), all used at 1:10,000 dilution. The background-corrected intensities of bands were calculated using Odyssey imaging system (LI-COR) as previously described [24].

### 2.6. Cellular Fractionation

RPE1 cells were fractionated using Qproteome Mitochondria Isolation Kit (Qiagen) according to manufacturers instruction. Briefly, cells incubated in a Lysis buffer were centrifuged at 1000×g for 10 min to produce a supernatant containing cytosolic proteins (Cy). The pellet was resuspended in Disruption buffer using a what gauge needle and re-centrifuged at 1000×g for 10 min to generate a pellet containing nuclei, cell debris, and unlysed cells (referred to as Nu/D). The supernatant was centrifuged at 6000×g for 10 min to pellet mitochondria (Mi) leaving the microsomal fraction in the supernatant (Mt). Both pellets were re-extracted in lysis buffer.

### 2.7. Protein Expression in Bacteria

All His-tagged fusion proteins were expressed as previously described [28] in *E. coli* BL21 (DE3), induced by 0.5 mM IPTG for 2 h and were collected by centrifugation. Equal amount of uninduced and induced cell pellets were extracted with 2X SDS-PAGE sample buffer using freezing-thawing cycle, and the crude lysates were used for testing the specificity of VDAC antibodies.

### 2.8. Statistical Analysis

Statistical comparisons were performed using unpaired (two sample equal variance) two-tailed Student’s *t*-Test in Excel (Microsoft). The significance (*p* value) of the differences between two groups are indicated as follows: *** indicates *p* < 0.0005, ** indicates *p* < 0.005, * indicates *p* < 0.025, and non-significant or n.s. indicates *p* > 0.025.

## 3. Results and Discussion

### 3.1. VDAC1 and VDAC2 Are Present in a Cellular Fraction Devoid of Mitochondria

Several studies found VDAC1 and VDAC2 at non-mitochondrial locations. We recently identified VDAC3 in a non-mitochondrial sub-cellular fraction and also found VDAC3 to localize at centrosomes [28]. To verify whether VDAC1 and VDAC2 are present outside mitochondria, and to compare the distribution of these two VDACs in cellular compartments with that of VDAC3, we performed a sub-cellular fractionation of RPE1 cells. The VDAC3 antibody used in our previous studies robustly detected recombinant VDAC3 by immunoblotting but did not significantly cross-react with recombinant VDAC1 and VDAC2 expressed in bacteria (Figure S1 and [28]). Similarly, antibodies against VDAC1 and VDAC2 strongly detected recombinant VDAC1 and VDAC2, respectively, and showed only marginal cross-reactivity to other VDACs (Figure S1).

**Figure 1:**
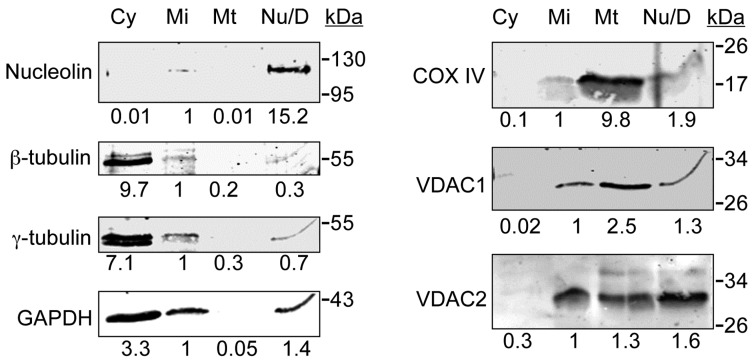
VDAC1 and VDAC2 are present in both mitochondrial and non-mitochondrial sub-cellular fractions. Asynchronously growing RPE1 cells were separated into cytosolic (Cy), microsomal (Mi), and mitochondrial (Mt) fractions. The pellet fraction that contained nuclei, debris and unlysed cellular components was further extracted in lysis buffer (Nu/D). Equal amounts (5 µg) of proteins from these sub-cellular fractions were separated on SDS-PAGE, transferred to nitrocellulose membrane and probed with antibodies against indicated proteins. Molecular weight standards (kDa) are shown next to the immunoblots. The numbers below each immunoblot represent the ratio of background-corrected intensities in each sub-cellular fraction to that in the microsomal fraction for the corresponding protein.

The sub-cellular fractionation method we used separates mitochondria from cytosolic, nuclear and microsomal compartments. We found that VDAC1 and VDAC2 were almost completely absent in the cytosolic fraction, and like Cytochrome Oxidase IV (CoxIV), were enriched in the mitochondrial fraction (Figure 1). However, like VDAC3 [28], a significant amount of VDAC1 and VDAC2 were also present in the microsomal fraction that also contains centrosomal proteins such as γ-tubulin but is largely devoid of mitochondria (Figure 1). The numbers below the blots in Figure 1 represent the relative intensity of each band compared to the microsomal fraction (normalized at 1.0), as determined using the LI-COR Odyssey imaging system. Given that the 5 µg of each fraction loaded on the blot represents 1.7% of the yield of the microsomal fraction and 3.2% of the yield of the mitochondrial fraction, this leads to the estimation that roughly 43% of VDAC1 and 59% of VDAC2 are found outside of mitochondria. Thus, combined with our previous study [28], this data suggests that a large fraction of all three VDACs are present outside mitochondria. While VDAC1 and VDAC2 are also present in the Nu/D fraction, we do not take this as support for nuclear localization of VDAC proteins, due to the presence of debris and unlysed cells in this fraction, which is only shown to demonstrate removal of nuclei from the other fractions.

### 3.2. VDAC1 and VDAC2 Localize to Centrosomes

**Figure 2:**
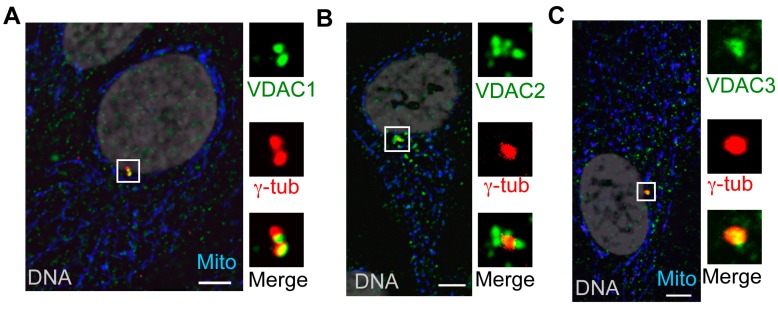
All three VDACs localize to centrosomes. (**A**–**C**) Representative images of asynchronously growing human RPE1 cells stained for γ-tubulin (γ-tub; red), mitochondria (Mito, blue), and (**A**) VDAC1 (green); (**B**) VDAC2 (green); and (**C**) VDAC3 (green). DNA is stained by Hoechst (gray). In this and other figures, panels show four-fold digitally magnified images of a region of interest, in this case surrounding the centrosomes. Bar = 5 μm.

Although we found VDAC3 to localize at centrosomes in addition to mitochondria, it was not clear whether this made VDAC3 unique among the VDACs, and we sought to determine if VDAC1 and VDAC2 also localize to centrosomes. When we used the specific antibodies against each VDAC to stain asynchronously growing human RPE1 cells, we noticed that, similar to the centrosomal localization of VDAC3 (Figure 2, and [28]), VDAC1 and VDAC2 were also found in the vicinity of centrosomes (as judged by co-localization with the centrosome marker γ-tubulin) (Figure 2). All VDAC antibodies also generated relatively weak cytoplasmic puncta that are often co-localized with mitochondria, though mitochondrial co-staining by VDAC2 antibody was more prominent than the other two (Figure 2). While centrosomal staining by VDAC1 antibody was co-localized on one or both γ-tubulin foci, VDAC2 seemed to predominantly localize at intensely stained foci surrounding centrosomes (Figure 2). VDAC1 and VDAC2 antibodies generated similar signals in other cell types (data not shown).

### 3.3. VDAC1 Predominantly Localizes to Mother Centrioles, While Centrosome-Associated VDAC2 Localizes to Centriolar Satellites

**Figure 3:**
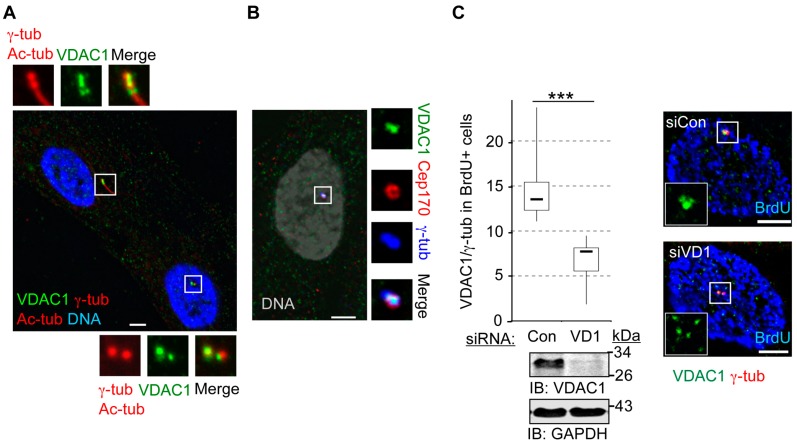
VDAC1 is more strongly associated with mother centriole. (**A**–**B**) Representative images of asynchronous RPE1 cells stained for VDAC1 (green), and (**A**) γ-tubulin (γ-tub; red) and Ac-tubulin (Ac-tub; red); (**B**) Cep170 (red) and γ-tub (blue). Ac-Tub stained centriolar microtubules and primary cilia. DNA is blue in (**A**) and grey in (**B**). Bar = 5 μm. (**C**) Asynchronously growing RPE1 cells treated with siRNAs against Lamin A/C (siCon) and VDAC1 (siVD1) for 72 h. Cells were labeled with BrdU, and stained for VDAC1 (green) and γ-tub (red). The box and whisker diagram shows the normalized fluorescence intensity values of centrosomal VDAC1 from twenty five cells, where boxes represent lower and upper quartiles, the marker in the box (-) indicates the median, and the whiskers represent minimum and maximum values. *p* value is derived from unpaired *t*-test. *** indicates *p* < 0.0005. Representative immunoblots at the bottom of the graph show depletion (roughly 80%) of whole-cell VDAC1 in siVDAC1 cells compared to control cells, where GAPDH is loading control. Molecular weight standards (kDa) are shown next to the immunoblots. Micrographs show representative siCon and siVDAC1 cells stained for VDAC1, γ-tub and BrdU (blue), Bar = 5 μm.

Although the VDAC1 antibody stained all centrioles present, the centriolar staining was often asymmetrical (Figure 3A), and VDAC1 was localized comparatively strongly at one of the centrioles in the majority of asynchronous RPE1 cells, regardless of the presence of primary cilia. Further localization experiments indicated that the predominant centrosomal staining of VDAC1 was concentrated at mother centrioles (Figure 3B) in roughly 50%–60% asynchronously growing cells, as judged by the co-labeling of cells with a Cep170 antibody [31]. Using a VDAC1-specific siRNA (siVDAC1), we are able to consistently deplete more than 80% of total VDAC1 protein in siVDAC1 cells, compared to control cells (siControl, Figure 3C). siVDAC1 also reduced the γ-tubulin-normalized centrosomal level of VDAC1 more than two-fold (Figure 3C), as determined by our modified quantitative immunofluorescence assay [30]. To ensure we compared centrosomal VDAC1 levels between cells at similar stages of the cell cycle, we restricted the analysis to cells that were in S-phase as judged by incorporation of BrdU; S-phase also represents the strongest VDAC1 signal at centrosomes (see below). Thus, the ability of siVDAC1 to reduce the strongest VDAC1 staining further verifies centrosomal localization of VDAC1. The gold standard for demonstration of a centrosomal pool of VDAC1 would be immuno-Electron Microscopy (EM). Unfortunately, the VDAC1 antibody we used here did not stain either mitochondria or centrioles in immuno-EM, probably due to incompatibility with the fixation method required for EM, and another VDAC antibody that we have tested does stain centrosomes but detects all three VDAC proteins (Figure S1B,C).

**Figure 4:**
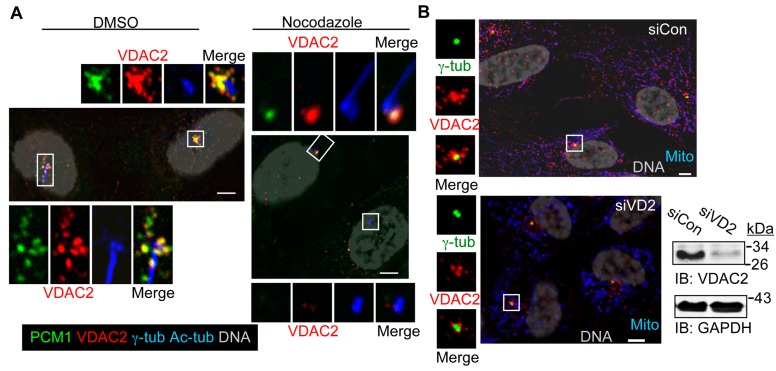
Centrosomal VDAC2 localizes to centriolar satellites. (**A**) Asynchronously growing RPE1 cells were treated with 2 μM Nocodazole or 0.05% DMSO for 4 h, fixed and stained for the PCM1 marker of centriolar satellites (green), VDAC2 (red), and Ac-tub plus γ-tub (both are blue); (**B**) Asynchronously growing RPE1 cells treated with control (siCon) or a VDAC2-specific siRNA (siVD2) for 72 h. Micrographs show random fields from the indicated populations of cells stained for γ-tub (green), VDAC2 (red) and mitochondria (Mito, blue) from identical imaging conditions. DNA is gray and bar is 5 μm in (**A**–**B**). Representative immunoblots shows depletion (roughly 70%) of whole-cell VDAC2 in siVDAC2 cells compared to control cells, where GAPDH is loading control. Molecular weight standards (kDa) are shown next to the immunoblots.

In contrast to VDAC1 (and VDAC3 [28]), centrosome-associated VDAC2 was instead predominantly found at foci surrounding centrosomes. This staining pattern is reminiscent of centriolar satellites [32], and VDAC2 strongly co-localizes with the centriolar satellite marker PCM1 in the vicinity of centrosomes in RPE1 cells (Figure 4A). The distribution of VDAC2 and PCM1 around centrosomes were significantly disrupted by a brief treatment with nocodazole (Figure 4A, Figure S2). This observation further indicates that VDAC2 localizes to centriolar satellites that depend on the microtubule cytoskeleton for their organization [33]. However, some residual staining of both PCM1 and VDAC2 was detected in roughly 15%–20% cells after nocodazle treatment (Figure 4A, Figure S2). In almost all cases, the residual staining was localized to centrioles, suggesting the presence of centriolar pools of these proteins that are not significantly affected by nocodazole. Thus, our results indicate that centrosomal VDAC2 predominantly localizes to centriolar satellites, and to some extent to the centrioles themselves. Treating cells with a VDAC2-specific siRNA (siVDAC2 or siVD2) that depleted cellular VDAC2 by roughly 70% compared to control cells greatly reduced the mitochondrial VDAC2 staining, and moderately diminished the centrosome-associated localization of VDAC2 (Figure 4B).

Though diminished to varying degrees, the centrosome-associated signals of all three VDACs were not completely lost upon respective siRNA treatment (Figure 3 and Figure 4, [28]). This may be due to incomplete knockdown of VDAC proteins, or higher stability of their centrosomal pools compared to that present at other sub-cellular compartments including mitochondria. Importantly, the centriolar pools of several centriolar proteins are resistant to siRNA-mediated depletion [34,35,36]. However, we cannot completely rule out the possibility that residual centrosomal signals reflect the modest cross-reactivity of VDAC antibodies that we observed *in vitro*. Nevertheless, our observations suggest that there are pools of both VDAC1 and VDAC2 that, similar to VDAC3, are localized to centrosomes or centrosome-associated sites.

### 3.4. Depletion of VDAC1, but Not VDAC2, Leads to Cilia Assembly in Non-Starved Cells

**Figure 5:**
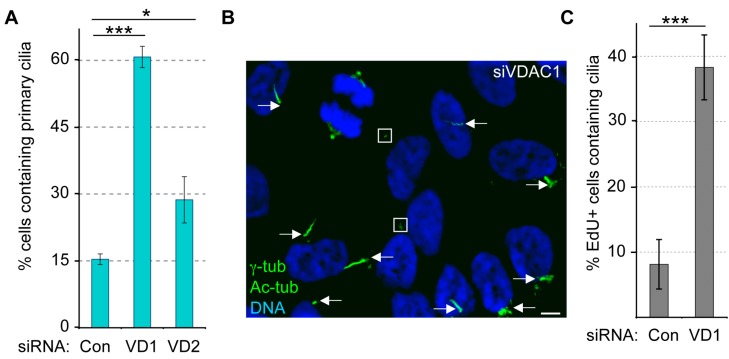
Depletion of VDAC1 leads to primary cilia assembly in non-starved cells. (**A**–**B**) Asynchronously growing RPE1 cells treated with siRNAs against Lamin A/C (Con), VDAC1 (siVDAC1 or VD1) and VDAC2 (VD2) were stained for γ-tub and Ac-tub to identify centrosome and primary cilia respectively. (**A**) Percentage of cells with cilia was plotted as bars where values represent mean ± SD for three independent experiments, 250–300 cells were counted per replicate. *** indicates *p* < 0.0005, * indicates *p* < 0.025; (**B**) Micrograph shows a random field of siVDAC1 cells stained for γ-tub and Ac-tub (both are green). Arrows mark cilia and squares surround centrosomes of non-mitotic cells that don’t have cilia; (**C**) Cells prepared as in (**A**) were labeled with EdU for 4 h. Bar graph shows the percentage of EdU-positive cells with Ac-tub stained cilia. Values represent the mean ± SD for three independent experiments where at least 100 cells were counted per replicate. *** indicates *p* < 0.0005.

One of the functions of the centrosomal pool of VDAC3 is to negatively regulate ciliogenesis [29]. Given our identification of centrosomal pools of VDAC1 and VDAC2, we next asked if these two proteins also regulate ciliogenesis. Generally, the ciliogenesis program in RPE1 cells is induced upon serum starvation, and the majority of RPE1 cells (85%–90%) assemble cilia after 48 h of serum starvation. While only 10%–15% of asynchronously growing RPE1 cells assemble cilia, roughly 65% of asynchronously growing siVDAC1 cells formed cilia as judged by staining for acetylated tubulin (Ac-tub) (Figure 5A,B). We observed a similar trend with another marker for the ciliary axoneme, polyglutamylated tubulin (data not shown). Ectopic expression of an siRNA-resistant (sir) version of VDAC1 (GFP-sirVDAC1) significantly reduced the number of cells with cilia as compared to the expression of GFP alone (roughly 1.7-fold; see below). Ectopically expressed GFP-sirVDAC1 localizes to centrosomes only in a sub-population of GFP-positive RPE1 cells (see below), and this may account for the observation that it does not restore ciliogenesis in siVDAC1 cells to levels seen in control cells (siControl expressing GFP alone). Nevertheless, the significant reduction of ciliogenesis phenotype in asynchronously growing siVDAC1 cells upon expression of GFP-sirVDAC1 indicates that the aberrant ciliogenesis phenotype in RPE1 cells is due to depletion of the VDAC1 protein. Importantly, the ciliogenesis phenotype in siVDAC1 cells was similar to that seen in siVDAC3 cells [29]. In contrast, depletion of VDAC2 caused only a moderate increase in cells with primary cilia compared to control cells (Figure 5A).

Next, we asked if this increase in cells with cilia upon VDAC1 depletion arose because these cells exited the cell cycle, or because they inappropriately formed cilia during interphase. To test this, we examined VDAC1-depleted cells that are in S-phase (as judged by incorporation of EdU during a 4 h pulse) for the presence of cilia. Interestingly, we observed a four-fold increase in EdU-positive siVDAC1 cells with cilia, compared to EdU-positive siControl cells (Figure 5C). The majority of cilia (roughly 85%) in asynchronously growing EdU-positive siVDAC1 cells contained Arl13b (Figure S3), an essential component of ciliary membrane [37], suggesting that majority of cilia that are formed inappropriately due to depletion of VDAC1 are mature. However, siVDAC1 cells undergoing mitosis lack cilia (Figure 5B), indicating that the cilia that were present in S-phase were ultimately disassembled before mitosis.

Nonetheless, VDAC1 depletion likely did cause a delay in cell cycle progression, as just roughly 10%–12% of asynchronously growing siVDAC1 cells incorporated EdU during a 4 h EdU pulse, compared to 33%–35% of siControl cells. This decrease in EdU incorporation may result from a drop in mitochondrial bioenergetics in siVDAC1 cells, a delay in S-phase entry due to the presence of cilia [38,39], or both. In order to monitor the overall mitochondrial function in individual VDAC-depleted RPE1 cells, we examined the ability of asynchronously growing cells treated with siRNAs to incorporate MitotrackerRed as a measure of membrane potential. While all control cells incorporated MitotrackerRed equally, 10%–15% of siVDAC1 cells displayed a moderate decrease in MitotrackerRed incorporation compared to siControl cells, whereas roughly 50% of siVDAC2 cells showed a significant loss in membrane potential (Figure S4). The decrease in membrane potential upon depletion of VDAC2 was previously noticed in VDAC2 knock-out MEFs [40]. MitotrackerRed incorporation in siVDAC3 cells was very similar to control cells (Figure S4), consistent with observations from VDAC3 knock-out MEFs [41].

Another study showed roughly 30%–40% loss of membrane potential upon depletion of VDAC1 in tumor-derived cells [42]. While this could reflect differences in depletion efficiency (that study used a higher siRNA concentration than used here), or cell type differences, it seems unlikely that a modest decrease in membrane potential can account for the observed ciliogenesis phenotype. In addition, even considering the important roles of VDAC1 at the OMM, it has been demonstrated that any of the three VDACs was dispensable, possibly due to compensation by the other two VDACs [41]. Importantly, we have shown that disrupting gross mitochondrial bioenergetics did not cause an aberrant ciliogenesis phenotype [29]. Overall, our data suggests that VDAC1 depletion, similar to that of VDAC3, leads to aberrant ciliogenesis in an asynchronous population of cells, and this effect is unlikely to be explained solely by cell cycle exit or loss of mitochondrial function. Instead, our data indicates that VDAC1 depletion initiates an inappropriate ciliogenesis program, or impairs the disassembly of cilia early in the cell cycle, either of which might in turn cause a delay in S-phase entry.

### 3.5. VDAC2 Promotes Cilia Maturation

**Figure 6:**
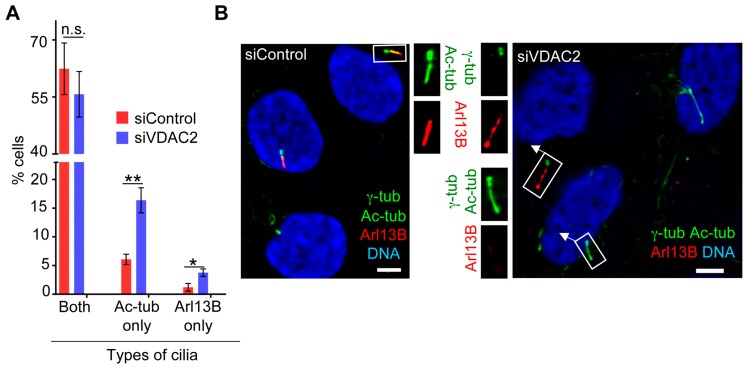
VDAC2 depletion causes subtle but significant defects in cilia assembly during 24 h serum starvation. (**A**–**B**) RPE1 cells treated with siControl or siVDAC2 were serum starved for 24 h, fixed and stained for γ-tub, Ac-tub (to mark ciliary axoneme) and Arl13B (to mark ciliary membrane). (**A**) Percentage of cells with cilia that were positive for both markers (both), or that were positive for only one marker (Ac-tub only or Arl13B only) were plotted as bars. Values represent mean ± SD for three independent experiments, where at least 200 cells were counted per replicate. ** indicates *p* < 0.005, * indicates *p* < 0.025, and n.s. indicates non-significant (*p* > 0.025); (**B**) Micrographs show representative fields of siControl and siVDAC2 cells stained for Arl13B (red), γ-tub, and Ac-tub (both are green). DNA is blue and bar = 5 μm.

Most of the proteins that localize to centriolar satellites regulate ciliogenesis either through regulating the transport of ciliary components to basal bodies or through vesicle trafficking [43]. Based on our observation that VDAC2 is localized to centriolar satellites, we anticipated that VDAC2 depletion might attenuate cilia assembly. Therefore, we examined cilia formation in control and VDAC2-depleted cells after 24 h of serum starvation, a time that when a significant fraction of cells might still be in the early stages of cilia assembly, to facilitate identification of defects in ciliogenesis. We did not observe any significant change in the percentage of cells that had assembled cilia, as judged by the presence of one of the cilia markers Ac-tub or Arl13B. However, when the cilia were further categorized, we observed that roughly 15% of siVDAC2 cells had a cilium that had a ciliary axoneme (Ac-tub positive) but lacked the ciliary membrane marker Arl13B, compared to 6% of control cells (“Ac-tub only”; Figure 6A,B). In addition, a small but distinct fraction of siVDAC2 cells (roughly 4%) had an apparent cilium that was positive for Arl13B but lacked the ciliary axonemal marker Ac-tub (“Arl13B only”; Figure 6A,B), a phenotype that was rarely seen in control populations. While it will likely require live cell imaging to elucidate the precise role of VDAC2, this observation suggests that VDAC2 may play a role in early stages of cilia assembly. A similar role was recently documented for the Joubert syndrome protein CSPP1, whose depletion in zebrafish led to a reduction in recruitment of Arl13B to the cilliary membrane despite the presence of an apparently normal ciliary axoneme [44]. Such a role for VDAC2 in maintaining ciliary membrane integrity is also supported by a recent siRNA screen that identified VDAC2 as a positive regulator of Hedgehog signaling [45]. Regardless, our data suggests that the function of VDAC2 at the centrosome/basal body is distinct from that of VDAC1 and VDAC3.

### 3.6. Ectopically Expressed VDAC1 and VDAC2 Localize to Centrosomes, and Overexpression of VDAC1 Suppressed Ciliogenesis upon Serum Starvation

**Figure 7:**
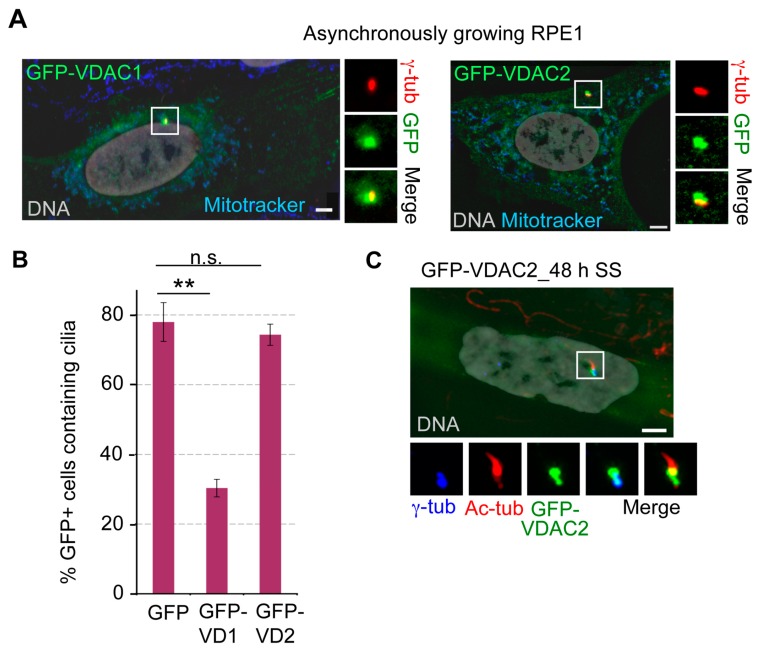
Overexpression of GFP-VDAC1 but not GFP-VDAC2, suppresses cilia formation during serum starvation. (**A**) Representative images of asynchronously growing RPE1 cells expressing GFP-VDAC1 or GFP-VDAC2 (green) that incorporated MitotrackerRed (red) and stained for γ-tub (red); (**B**–**C**) RPE1 cells expressing GFP-VDAC1 or GFP-VDAC2 were serum starved for 48 h, stained for Ac-tub and γ-tub, and the percentage of GFP-positive cells with primary cilia was counted. Values represent the mean ± SD for three independent experiments where 75–100 cells were counted per replicate. ** indicates *p* < 0.005, and n.s. indicates non-significant (*p* > 0.025); (**C**) In roughly 10%–12% of serum starved GFP-VDAC2 expressing cells, GFP signal is predominantly localized at a region between basal body and ciliary axoneme, suggesting possible localization of GFP-VDAC2 at transition zones. Shown is a representative cell expressing GFP-VDAC2 (green), and stained for Ac-tub (red) and γ-tub (blue). In both (**A**) and (**C**), DNA is grey, bar = 5 μm.

We found that etopically expressed GFP-VDAC1 and GFP-VDAC2 were localized to centrosomes in about 40%–45% and 25%–30% of asynchronously growing RPE1 cells, respectively (Figure 7A). Additionally, both these GFP-tagged VDACs were diffusely cytosolic and were also nicely co-localized with mitochondria (as judged by co-localization with MitotrackerRed) in roughly 30% and 50% of RPE1 cells, respectively.

Consistent with the fact that VDAC1 depletion leads to cilia formation, ectopic expression of GFP-VDAC1 inhibited cilia assembly during serum starvation. While almost 75% of RPE1 cells expressing GFP alone formed cilia after 48 h of serum starvation, only roughly 30%–35% of cells expressing GFP-VDAC1 could form cilia under this condition that supports ciliogenesis (Figure 7B). This result supports the hypothesis that like VDAC3, VDAC1 negatively regulates ciliogenesis. Notably, the percentage of cells ectopically expressing GFP-VDAC2 with cilia was similar to that in cells expressing GFP alone, further confirming that the function of VDAC2 in ciliogenesis is distinct from that of either VDAC1 or VDAC3 (Figure 7B). Moreover, in roughly 10%–15% of GFP-positive cells, centrosome-associated GFP-VDAC2 signal was predominantly localized at the base of the cilia or transition zone (Figure 7C), a localization not seen in cells expressing GFP-VDAC1 or GFP-VDAC3 [29]. Notably, mouse VDAC2, but not VDAC1 or VDAC3, was identified in two separate proteomic analyses of complexes of NPHP2 [9], and B9D1 [46]. It is tempting to speculate that GFP-VDAC2 may associate with a transition zone protein such as NPHP2 that localizes to both the basal body and transition zone, particularly when cells form cilia.

### 3.7. The Roles of VDAC1 and VDAC3 in Ciliogenesis are Non-Redundant

**Figure 8:**
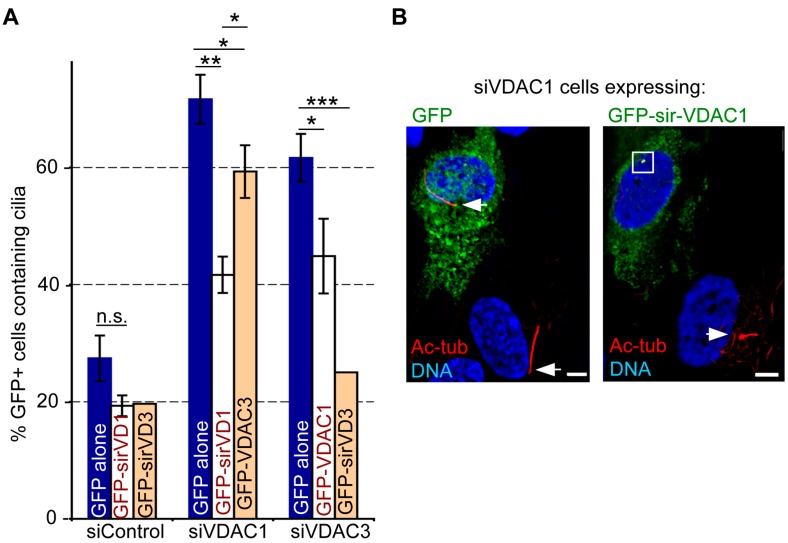
Overexpression of VDAC1 and VDAC3 only marginally rescues ciliogenesis in VDAC3- and VDAC1-depleted cells. (**A**) Asynchronously growing RPE1 cells treated with control, siVDAC1 and siVDAC3 [29] were subsequently transfected with GFP, GFP-VDAC1 or GFP-sirVDAC1 and GFP-VDAC3 or GFP-sirVDAC3. Cells were stained for Ac-tub and γ-tub, and the percentage of GFP-positive cells with Ac-tub labeled primary cilia was counted, and plotted as bars. Values represent the mean ± SD for three independent experiments where 75–100 cells were counted per replicate, with the exception of siControl and siVDAC3 cells expressing GFP-sirVDAC3, a previously published result [29] for which we counted only a single replicate. *** indicates *p* < 0.0005, ** indicates *p* < 0.005, * indicates *p* < 0.025, and n.s. indicates non-significant (*p* > 0.025). Note that because the result is previously published [29], we performed only a single replicate for siVDAC3/GFP-sirVDAC3, and the designation of highly significant (***) refers to a statistical analysis of the previously published data; (**B**) Micrographs show representative images of siVDAC1 cell population transfected with constructs expressing GFP alone and GFP-sirVDAC1. Cells were stained for GFP (green) and Ac-tub (red). DNA is blue, bar = 5 μm. Arrows mark cilia, and the white box in siVDAC1 cell expressing GFP-sirVDAC1 surrounds a GFP-positive centrosome that did not have a cilium.

Because the aberrant cilia formation in asynchronously growing siVDAC1 cells was similar to what we reported previously in siVDAC3 cells [29], we next sought to determine if VDAC1 and VDAC3 perform redundant functions to negatively regulate ciliogenesis. Therefore, we tested whether ectopic expression of VDAC3 can reverse the aberrant ciliogenesis in asynchronously growing siVDAC1 cells, and vice versa. Overexpression of a siRNA-resistant version of VDAC3 (GFP-sirVDAC3) led to a highly significant (*p* < 0.0005 as determined using our previously published data [29]) reduction in the percentage of siVDAC3 cells that had cilia (Figure 8A), indicating that a GFP-tagged version of VDAC3 can rescue the ciliogenesis phenotype caused by VDAC3 depletion. In comparison, GFP-tagged VDAC1 (GFP-sirVDAC1) appeared to be only partially active in this assay, but still partially rescued the ciliogenesis phenotype caused by VDAC1 depletion and led to a significant (*p* < 0.005) reduction in the percentage of VDAC1-depleted cells that had cilia (Figure 8A,B). However, GFP-VDAC3 could not reverse ciliogenesis in siVDAC1 cells to the same extent as GFP-sirVDAC1, even though GFP-sirVDAC1 was less active in this assay; the difference between siVDAC1 cells expressing GFP and GFP-VDAC3 was only modestly significant (*p* < 0.025), and the difference between siVDAC1 cells expressing GFP-VDAC3 and GFP-sirVDAC1 remained significant (*p* < 0.005). Therefore, while GFP-VDAC3 can replace endogenous VDAC3 in this assay, it cannot effectively replace endogenous VDAC1. Similarly, the difference between siVDAC3 cells expressing GFP and GFP-VDAC1 was of only modest significance (*p* < 0.025) (Figure 8A). Because GFP-sirVDAC1 only partially suppressed the appearance of cilia in siVDAC1 cells, we cannot rule out the possibility that the presence of the GFP-tag compromises the function of VDAC1 and prevents it from completely rescuing the siVDAC1 phenotype or replacing endogenous VDAC3. However, because GFP-tagged VDAC1 caused a significant (*p* < 0.005) reduction in the percentage of siVDAC1 cells with cilia but had only a modest effect in siVDAC3 cells (*p* < 0.025), it seems more likely that VDAC1 cannot effectively replace endogenous VDAC3. Regardless, because GFP-VDAC3 clearly cannot replace endogenous VDAC1, this data suggests that the functions performed by VDAC1 and VDAC3 to suppress inappropriate cilia assembly in asynchronously growing cells are largely non-redundant.

### 3.8. VDAC1 Transitions from Centrosomes to Spindles during Mitosis

**Figure 9:**
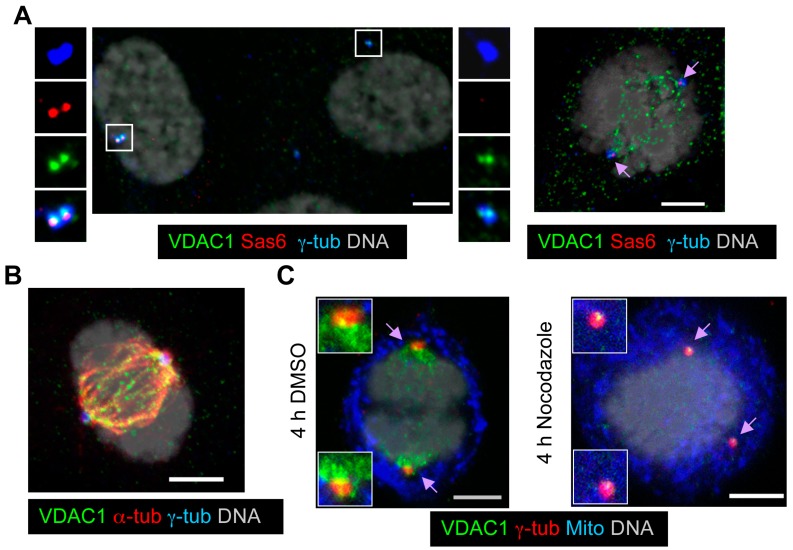
Centrosomal VDAC1 levels vary through the cell cycle, and VDAC1 transitions from centrosomes to spindles during mitosis. (**A**–**B**) Representative images of asynchronously growing RPE1 cells stained for γ-tub (blue), VDAC1 (green); and (**A**) Sas6 (red, marker for centriolar cartwheel); and (**B**) α-tub (red). Arrows in the top right image indicate poles of mitotic spindles; (**C**) Growing RPE1 cells briefly treated with nocodazole or DMSO were stained for VDAC1 (green), γ-tub (red) and mitochondria (Mito, blue). In (**A**–**C**), DNA is grey, and bar = 5 μm.

As discussed above, localization of VDAC1 at centrosomes varies during the cell cycle. We found that the centrosomal VDAC1 level was comparatively stronger in S-phase RPE1 cells; either judged by incorporation of BrdU or the presence of two distinct foci of Sas6 (Figure 9A). Sas6, a major component of centriolar cartwheels, is recruited to centrosomes during procentriole assembly and degraded during mitosis [47]. Additionally, during S-phase, only the mother centriole contains appendages, while the daughter centriole starts to accumulate appendage proteins such as hCenexin1/Odf2 as it matures during G2-phase (Figure S5). Using Sas6 and hCenexin1/Odf2 as markers for different phases of the cell cycle, we observed that centrosomal VDAC1 signal slowly decreases as cells pass from S-phase to mitosis, when it is almost lost from spindle poles (Figure 9B, Figure S5A). Instead, during mitosis VDAC1 is strongly localized on the spindle microtubules (Figure 9B). This localization of VDAC1 is completely lost when cells were briefly treated with nocodazole (Figure 9C), suggesting that the spindle localization of VDAC1 is likely mediated by its binding directly to microtubules, or a microtubule binding protein. The potential role of VDAC1 on the spindle is beyond the scope of this study. However, it may be important to note that VDAC1 interacts with Tctex-1 [48,49], a light chain subunit of cytoplasmic dynein that also localizes to spindle poles, minus ends of spindle microtubules, and kinetochores during mitosis [50] and negatively regulates ciliogenesis [51]. In addition, VDAC1 located at the OMM and ER were able to bind to free tubulin [52], microtubules and MAP4, a microtubule associated protein that stabilizes microtubules [53]. However, we verified that the spindle localization of VDAC1 is not due to alignment of mitochondria along the mitotic spindle (Figure 9C). Moreover, this mitotic spindle localization was not seen for VDAC2 or VDAC3, further highlighting the distinct molecular regulation and non-redundant functions of three VDACs at non-mitochondrial locations. Nevertheless, the data presented here suggests, in addition to their mitochondrial localization, all three VDACs localize at centrosomes/basal bodies and play important functions in regulating ciliogenesis.

## 4. Conclusions

Previously, we identified a non-mitochondrial pool of VDAC3 associated with centrosomes [28]. In this study, we demonstrated the presence of non-mitochondrial pools of the other two human VDACs, VDAC1 and VDAC2, and found these two VDACs also localize to centrosomes/basal bodies and centriolar satellites, respectively. Accordingly, we conclude that there is a centrosome-associated pool of each VDAC, in addition to the well known mitochondrial pool. Importantly, we discovered novel roles of these VDACs in ciliogenesis; VDAC1 and VDAC3 suppress cilia formation during asynchronous growth, while VDAC2 may support early stages of cilia assembly. These functions of VDAC proteins are likely due to their centrosomal localization, and not the well-established roles of VDACs in mitochondrial bioenergetics at the OMM. Our data also suggests that while VDAC1 and VDAC3 both negatively regulate ciliogenesis, their functions in ciliogenesis appear to be non-redundant.

Cilia are present in most animal cells either as flagella (unicellular eukaryotes and sperm), motile cilia (multiciliated epithelial cells or at the embryonic node) or sensory cilia (primary cilia) [3]. Although there are several studies identifying mechanisms of flagella assembly, IFT and ciliary beating [6,54], the importance of this tiny organelle was obscured for a long time. However, several discoveries in recent years demonstrated the stepwise assembly of cilia and the molecular architecture of ciliary compartments, and also elucidated the immense importance of both motile and primary cilia by identifying mutations in cilia-regulating genes and/or ciliary dysfunction in a wide range of diseases collectively known as ciliopathies [55,56,57]. Studies investigating ciliogenesis have identified several novel non-structural proteins at centrosomes that include mediators of vesicle transport and membrane-binding proteins [58,59,60,61]. In fact, in contrast to the historical notion of the centrosome as an exclusively non-membranous organelle, it was recently demonstrated that the centrosomes is constitutively associated with membrane components [62], as is required for a structure that must recruit vesicles containing ciliary cargo and dock with the plasma membrane in order to mediate ciliogenesis. Therefore, perhaps it should not be surprising to identify VDACs as components of centrosomes.

Moreover, there is ample evidence to suggest that mammalian VDACs are not exclusively found at the OMM [14]. VDAC1 was identified at the plasma membrane where it is localized to caveole [20,63,64], and interacts with the dynein-light chain Tctex1 [49] that negatively regulates ciliogenesis through a dynein-independent mechanism [51]. VDAC2 and VDAC3 are both present in the sperm outer dense fiber [23], a non-membranous component associated with the sperm flagella, and we previously documented a centrosomal pool of VDAC3 [28]. VDAC3 cooperates with Mps1 to suppress ciliogenesis [29], but it remains to be determined whether VDAC1 suppresses ciliogenesis in a similar manner. VDAC2 was also found in proteomic studies characterizing the ciliary NPHP and B9D1 complexes [9,46]. While the function of VDAC2 at cilia remains to be elucidated, our suggestion of a role in the initiation of ciliogenesis is consistent with the function of the NPHP complex, which is not required to build a cilium per se, but rather appears to be required for the assembly of specific signaling complexes that are found in cilia and regulate epithelial morphogenesis [9]. We speculate that VDAC2 is likewise required for recruitment to cilia of some subset of proteins or protein complexes that themselves are either not essential for ciliogenesis or can be recruited to cilia through other means.

However, the structural conformation of VDACs at centrosomal sites remains as an important open question. VDAC1 adopts an amphipathic beta-barrel structure, with hydrophobic surfaces interacting with the OMM and hydrophilic surfaces constituting the channel [15], and it is predicted that the other two VDACs adopt a similar conformation at the OMM [18]. While the historical view of centrosomes as non-membranous structures makes it difficult to envision this structure at centrosomes, centrosomes do in fact contain membranes [62]. Therefore, it is possible that VDACs also form beta barrel structures at centrosomes through interactions with centrosome-associated membranes. However, it is also possible that the structure of VDACs at centrosomes is distinct from that found in mitochondria. For example, the VDACs may adopt a more flexible structure at centrosomes that lacks channel activity but allows interactions with centrosomal proteins through hydrophilic surfaces and interaction with membranes through hydrophobic surfaces. An intriguing alternative, suggested by the observation that amphipathic beta structures can adopt either soluble or membrane-bound conformations [65], is that the VDACs may form inside out beta barrels at centrosomes, with hydrophilic surfaces facing outward to interact with centrosomal proteins. However, at this point, these assumptions are largely speculative, and while there may be no need to invoke a different structure, it will require detailed and extensive studies to elucidate the structural conformation of VDACs at non-mitochondrial sites such as centrosomes. We look forward to generating VDAC mutants that can accumulate at centrosomes but not mitochondria, in order to further explore the structure and function of centrosomal VDAC proteins.

## Supplementary Materials

Supplementary materials can be accessed at: http://www.mdpi.com/2073-4409/4/3/331/s1

## Abbreviations

siCon: Control siRNA
siVDAC1 or siVD1: VDAC1-siRNA
siVDAC2 or siVD2: VDAC2-siRNA
siVDAC3 or siVD3: VDAC1-siRNA
BrdU: 5-Bromo-2′-Deoxyuridine
Edu: 5-ethynyl-2′-deoxyuridine
