# Supplementary material for: Non-Overlapping Distributions and Functions of the VDAC Family in Ciliogenesis
Source: Cells. 2015 Jul 31;4(3):331–53. doi: 10.3390/cells4030331 (PMC4588040; doi:10.3390/cells4030331)
Supplement: Supplementary file 1 [file cells-04-00331-s001.pdf]

Supplementary

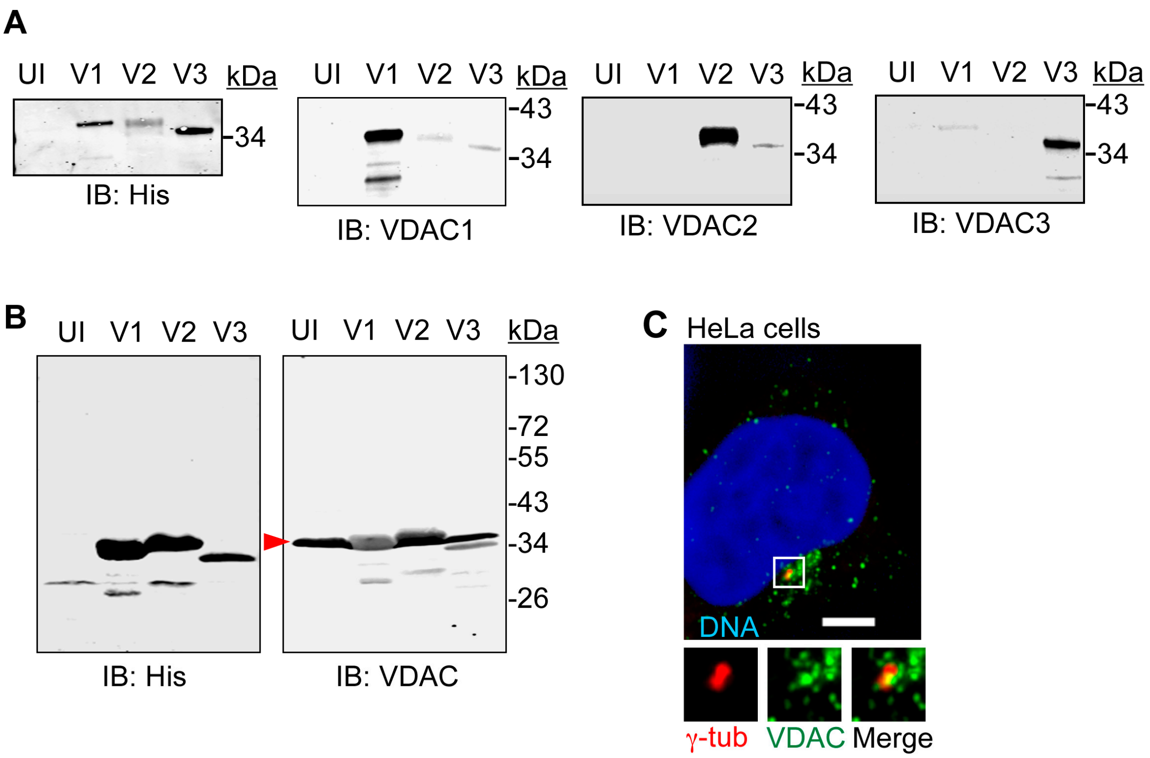

Figure S1. Antibodies against each VDAC show minimal cross-reactivity to the other two VDACs on immunoblots. (**A**–**B**) Crude lysates of uninduced (UI) and induced bacterial cells expressing 6XHis-VDAC1 (V1), 6XHis-VDAC2 (V2), and 6XHis-VDAC3 (V3) were immunoblotted with indicated antibodies. Molecular weight standards (kDa) are shown next to the immunoblots. (**A**) The anti-VDAC antibody (Cell Signaling) was raised against recombinant VDAC1 proteins, and is referred to as anti-VDAC1 antibody. Nearly equal amount of His-tagged proteins were loaded as analyzed by coomassie staining. All three antibodies detected their specific VDAC protein very strongly, and showed minimal cross-reactivity other two VDACs; (**B**) Samples were immunoblotted with anti-His antibody (also shown in [28]) and another anti-VDAC antibody (from Calbiochem) that reacted almost equally to each three His-VDAC protein. The red arrowhead indicates a non-specific protein detected by this anti-VDAC antibody; (**C**) Representative image of a S-phase arrested HeLa cell stained by antibodies against γ-tubulin (γ-tub; red), and VDAC (Calbiochem; green). DNA is blue and bar = 5 μm. Roughly 30%–40% of HeLa cells show VDAC staining at centrosomes in addition to cytoplasmic foci.

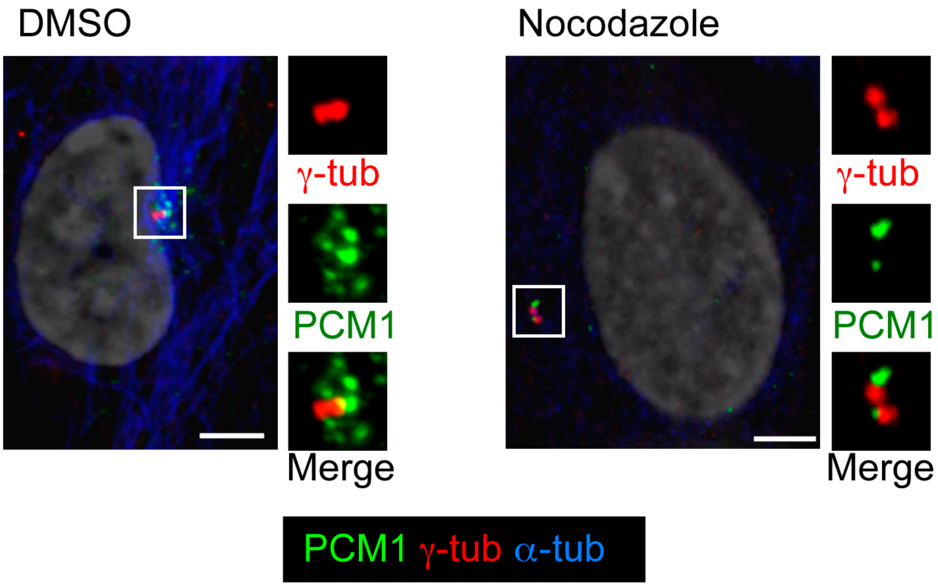

Figure S2. Nocodazole treatment disrupts the organization of centriolar satellites. Representative images of RPE1 cells prepared as in Figure 4 and stained for PCM1 (green, marker for centriolar satellites), γ-tub (red), and α-tubulin (α-tub, blue). Clearly, nocodazole treatment disrupted the microtubule network, and thereby led to disorganization of centriolar satellites as judged by PCM1 staining.

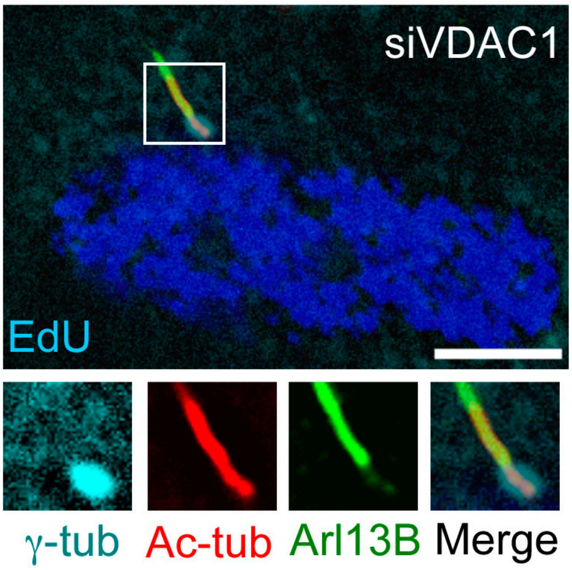

Figure S3. Inappropriately formed cilia in growing siVDAC1 cells contain ciliary membrane. Representative image of a siVDAC1 cell prepared as in Figure 5A, labeled with EdU (blue) for 4 h, and stained for γ-tub (cyan), Ac-tub (red) and Arl13B (marker for ciliary membrane; green). Bar is 5 μm.

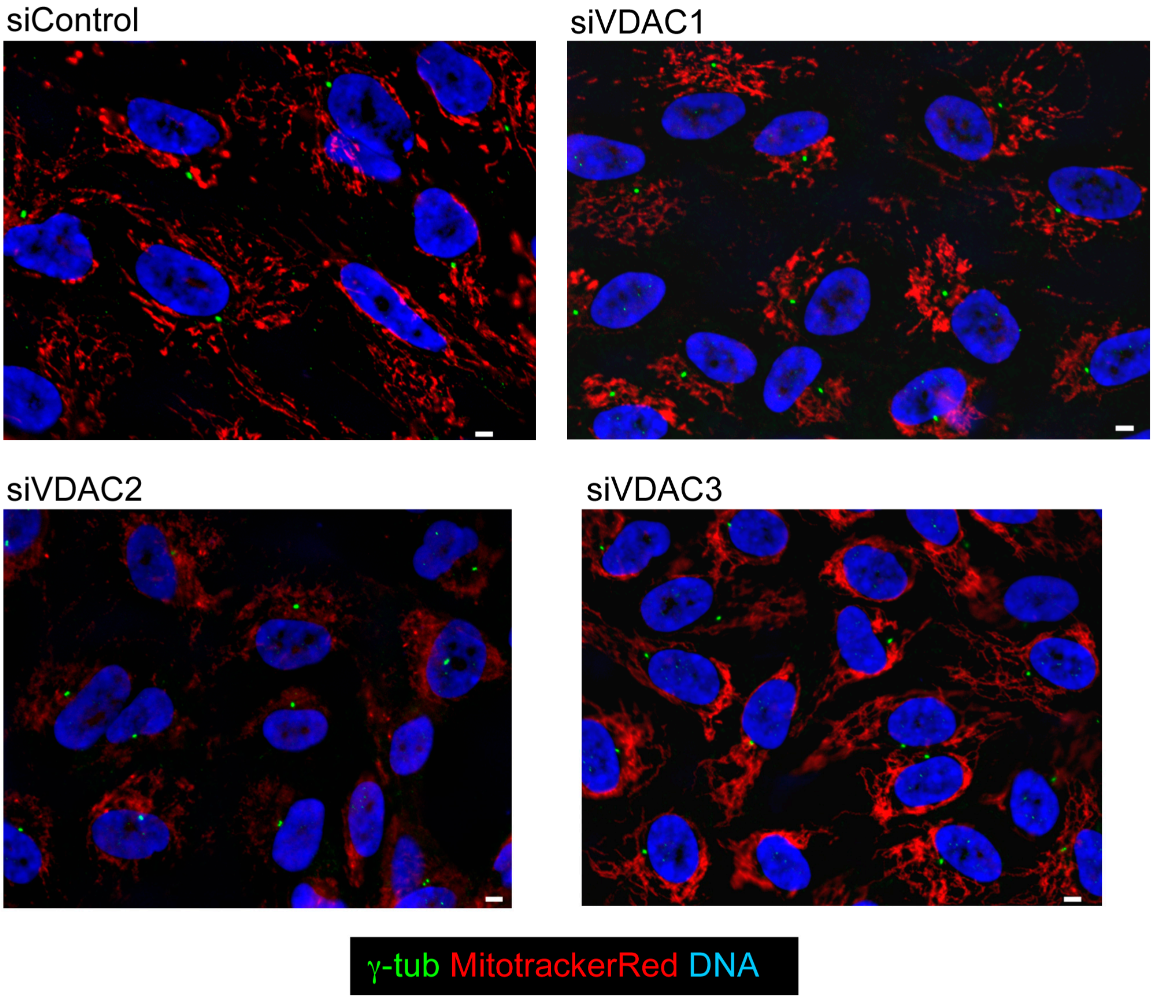

Figure S4. Depletion of VDAC2, but not VDAC1 or VDAC3 causes significant change in mitochondrial membrane potential in RPE1 cells. Asynchronously growing RPE1 cells treated with siRNAs against Lamin A/C (siControl), VDAC1 (siVDAC1), VDAC2 (siVDAC2) and VDAC3 (siVDAC3) were incubated with 100 μM MitotrackerRed (red) for 1 h, washed and grown in cell culture medium for 30 min. Cells were then fixed, and stained for γ-tub (green) and DNA (blue). Bar = 5 μm.

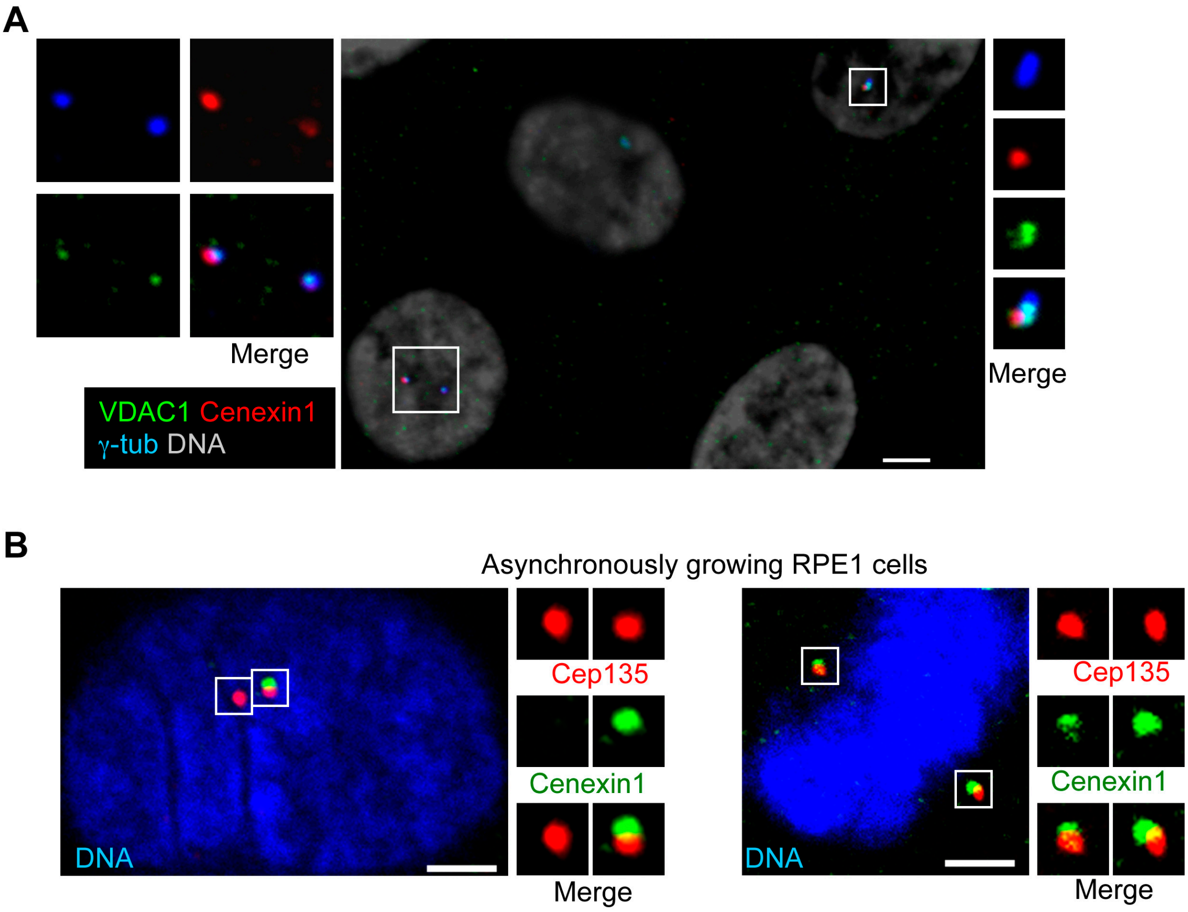

Figure S5. The centrosomal VDAC1 level varies in different cell cycle phases. (**A**–**B**) Representative images of asynchronously growing RPE1 cells stained for (**A**) γ-tub (blue), VDAC1 (green), and hCenexin1/Odf2 (red); and (**B**) hCenexin1/Odf2 (green) and Cep135 (red). In (**A**), the cell on the right is S-phase (as judged by one strong hCenexin1/Odf2 focus on one of the two γ-tub lobes) and the cell on the left is in late G2-phase (as judged by two separated centrosomes, with one having stronger and the other one having faint hCenexin1/Odf2 staining). DNA is grey in (**A**) and blue in (**B**), and bar = 5 μm.

Table S1. List of primers used in this study.

Primer Name	Purpose	Sequence
VDAC1 Fwd inf in GFP	Infusion primer to clone VDAC1 ORF into pHF286 to construct pHF307	5′-ACGAGCTGTACAAGGGTACCATGGCTGTGCCACCCACG-3′
VDAC1 Rev inf in pECE	Infusion primer to clone VDAC1 ORF into pHF286 to construct pHF307	5′-TACTTATCTATCTAGACTATTATGCTTGAAATTCCAG-3′
VDAC2 Fwd inf in GFP	Infusion primer to clone VDAC2 ORF into pHF286 to construct pHF308	5′-ACGAGCTGTACAAGGGTACCATGGCGACCCACGGACAG-3′
VDAC2 Rev inf in pECE	Infusion primer to clone VDAC2 ORF into pHF286 to construct pHF308	5′-TACTTATCTATCTAGACTATTAAGCCTCCAACTCCAG-3′
VDAC1 siRes mut top	Site directed mutagenesis of pHF307 to pHF309	5′-GTTTAGGATACACACAAACCCTTAAGCCAGGTATTAAAC-3′
VDAC1 siRes mut bottom	Site directed mutagenesis of pHF307 to pHF309	5′-GTTTAATACCTGGCTTAAGGGTTTGTGTGTATCCTAAAC-3′
